# President’s Annual Message

**Published:** 1905-01

**Authors:** A. W. Stirling

**Affiliations:** C.M. (Edin); D.P.H. (Lond.) Atlanta, Ga.


### PRESIDENT'S ANNUAL MESSAGE.*

BY A. W. STIRLING, M.D.; C.M. (EDIN.); D.P.H. (LOND.),
ATLANTA, GA.

GENTLEMEN: If you wish to know why I am standing here with a paper in my hand I must refer you to section 7, article 18 of the rules and regulations of our association, where you may find these words: "The president shall, at the close of his administration, at the regular meeting for the election of officers, submit to the society an annual message, devoted to an account of his stewardship and to the discussion of the interests, objects and business of the society."

This law was no doubt laid down with a kindly consideration for the president without thinking of his victims. The authors evidently had in mind the cuttlefish. I need hardly remind my learned colleagues that the cuttlefish has a pretty habit of ejecting in the faces of its neighbors an inky fluid, behind a black cloud of which he quietly steals away.

It is this inky fluid which I am now pouring out on you, but while the cloud may be thick, I promise you it will not be of great dimensions. For, what might with equal propriety be called the scuttlefish, has one advantage over me, it seems to have a never-failing supply of ammunition. *My* predicament keeps bringing into my head a certain Highland minister. As you are aware the Highlander in his native state expresses his emotions and other things in a language called the Gaelic. He learns English as you and I learn French and Spanish, and with a result sometimes as comical to those who understand it better. This special gentleman having to preach one Sunday in the language he had carefully acquired, began his discourse with the following words:

"My dearly beloved brethren, you will find the subject of our observations this afternoon in the First Epistle General of Peter, the fifth chapter and the eighth verse, and in these words: 'The devil as a roaring lion walketh about seeking whom he may devour.' Now, my brethren, with your leave we will divide the

*Read before the Atlanta Medical Society.

subject of our text into four heads. First, we shall endeavor to ascertain, who the devil he was. Secondly, we shall inquire into his geographical position, namely, where the devil he was going. Thirdly, and this is of a personal character, we shall ask ourselves, who the devil he was seeking. Fourthly, and lastly, my beloved brethren, we shall endeavor to solve a problem that has never been solved to this day, namely, what the devil he was roaring at."

I wonder, my beloved brethren, in the words of the good divine, "what the devil" I can say which will be for your edification. I am instructed to give an account of my stewardship, but my stewardship is before you all and no words of mine can now alter it. They would be too late. The year is finished and the best thing we can now do is to see that next year shall be a step in advance of this. We have knocked the vitals out of some twenty-six subjects, and no doubt with benefit to all of us; and discussed besides a considerable number of related cases. Happily in the interest of our future meetings there are still some diseases left upon the books.

We have seen a few pathological specimens, and some gentlemen have kindly induced patients to come for our inspection. But this is a department of the society which deserves more of our attention. I nominated a committee at the beginning of the year whose function it was to produce all sorts of cases and specimens. But either because the matter was insufficiently dwelt upon, or because it is not always an easy thing to find cases, or for some other reason, we have not been so well supplied in that way as I had hoped; and if I might take the liberty of making a suggestion to my successor, I think he would do well to make a strong point of this subject.

In general terms the society is fairly prosperous. Our worthy secretary, whose kind coöperation, good-nature and willingness I desire to record, will no doubt tell us what advance there may have been in our numbers; and our careful treasurer, whose health, I regret to say, has not permitted his attendance so frequently as we should all have liked, will inform us concerning our financial state. An increased membership is good for the society, and I think we may say for those who join it. There is still room for some improvement in the attendance; I hope that next year we shall have it.

Our object, short of which we must not come, should be to make the society so interesting, so instructive, and so important, that no self-respecting medical practitioner might feel that he could for a moment afford to be outside of it.

Committees have from time to time been appointed to deal with various matters as they came up. The most important was that to work with certain others in endeavoring to limit and finally eradicate tuberculosis, for which the society voted a gift of twenty-five dollars, to which was added a surplus nine from our recent supper.

One of the objects of our Society is the maintenance and increase of fellowship. With this in view, and also that our families might come to be at least on bowing terms, we had last month a supper at one of the hotels. I still think that the idea was good, and the attendance of both ladies and gentlemen was excellent, but owing to an unfortunately long wait, and perhaps to an incomplete knowledge among ourselves of what was expected, I fear we were perhaps rather more solemn than was absolutely essential. There are two rocks on which such meetings may go to pieces. It would be hardly in keeping with the dignity of our profession that we should allow our sense of fun too unlimited and uncontrolled a tether, and on the other hand a too funereal aspect is just about as bad. I hope we shall develop an annual social gathering which will have all the gaiety in the world without a sign of license.

One thing I was glad to see at our recent supper, though with some excusable surprise. I was glad to observe how deeply, if suddenly, the earnest teachings of our honored confrere, Dr. Kime, had sunk into the souls of nearly everybody present. Personally, I felt myself to be a bibulous sot in the midst of an exalted band of ascetic saints; and, had I not been so ably supported on either hand by church and state, I should also have felt like sinking beneath the table, not exactly from intoxication, but from an overpowering sense of my own wickedness.

In looking back over the past twelve months, all of us who are not quite new members must recall that this time last year Dr. Hardon was one of our most useful and instructive speakers, one whose regular attendance was an evidence of the interest he took in the Society. We still miss him, and deplore his loss.

There is little further to be said. If we have made any progress in advancing the "objects, interests and business" of the Society, it is not wise to dwell too much upon that aspect, for there is still room to improve. We want the combined thought and energy of all the members. If the next president desires to continue the methods used by myself, I am sure he will be grateful for your assistance during the following few days, in enabling him to arrange and print a programme before our next meeting; without your willing concurrence it would I know be a matter of difficulty, but I am certain that he will receive your help as I did.

It is almost necessary for the president of a society like this, under instructions to speak of its ends and objects, to make some reference to that higher aspect of our duties which, if we forget, we shall not merit the verdict of "Well done," when, in the relentless course of nature, we are forced to lay aside all thoughts of diagnosis and prognosis, and are ourselves the objects of the anxious treatment of our colleagues. It is not for me to preach a sermon on this or any other topic, but you perhaps will not object to listening to a few lines which a friend sent me the other day, probably because she knew how much I required to be reminded of their sentiment. They were written by Robert Louis Stevenson, not looked upon usually as a poet, but blessed with a great sense of the fitness of things, for he admired doctors far above lawyers and artists and millionaires and all such common folks as these. And he ought to have known, for he was the nephew of George Balfour, recognized in his day as a great authority on diseases of the heart. Through him Stevenson came in contact with many of our kind. Stevenson called his little poem "The Celestial Surgeon," and in it he drew a parallel between our profession and the Divinity himself (which, when we consider some people's gods, may with propriety be termed a compliment to both). In it he points out the stern necessity of being "cruel to be kind," for the character's sake as well as for the body's. He said:

If I have faltered more or less
In my great task of happiness;
If I have moved among my race
And shown no glorious morning face;
If beams from happy human eyes
Have moved me not; if morning skies,
Books, and my food, and summer rain
Knocked at my sullen heart in vain:—
Lord, thy most pointed pleasure take,
And stab my spirit broad awake;
Or, Lord, if too obdurate I,
Choose thou, before my spirit die,
A piercing pain, a killing sin,
And to my dead heart run them in!

I can not close my remarks without tendering to the members of the Society, both as a body and individually, my very sincere thanks for the kindly and courteous manner in which they have received my poor attempts to preside, and for the very generous way they have supported every effort for the Society's good. I am thankful to say there has been no difficulty in connection with parliamentary procedure, no hard question has had to be solved, and especially that Dr. Lindorme's gavel, his kind and weighty present to the society, has never once been called in to decide disputes by the cruel art of war.

I wish especially to lay stress upon the loyal way in which the members as a whole have followed up the idea of a programme for the whole year. In the necessary hurry of arranging it so as to be in time for the first meeting of the year, it was not possible to consult the majority of those asked to write concerning their preference in the matter of subject; and they were merely informed of its name and invited to indite a paper for a certain date. I believe every one who agreed intended to write, and very few failed us when the time came, and then usually for good and sufficient reasons. We have had not one barren meeting, for which we have to thank the general good will of the members.

I again heartily thank you for your consideration.

